# High-density mapping of his-ventricular intervals: impact of recording site and sex-specific differences

**DOI:** 10.1007/s10840-026-02438-z

**Published:** 2026-09-18

**Authors:** Teodor Serban, Jeanne du Fay de Lavallaz, Sven Knecht, David Spreen, Behnam Subin, Reto Stump, Philipp Krisai, Nicolas Schärli, Beat Schär, Felix Mahfoud, Christian Sticherling, Michael Kühne, Patrick Badertscher

**Affiliations:** 1 University Hospital of Basel, University of Basel, Petersgraben 4, 4031 Basel, Switzerland; 2 Cardiovascular Research Institute Basel, University of Basel, Basel, Switzerland

**Keywords:** His-Ventricle Interval, His Cloud, His Bundle Electrogram

## Abstract

**Background:**

Accurate assessment of the His–ventricular (HV) interval is essential to diagnosing infranodal conduction disease, but the influence of recording site and sex on HV values is not well defined.

**Objective:**

To evaluate the impact of measurement location and sex on HV interval values using high-density (HD) mapping catheters in patients undergoing atrial fibrillation (AF) ablation.

**Methods:**

We prospectively studied patients in sinus rhythm undergoing AF ablation. HV intervals were measured with a decapolar catheter and a HD mapping catheter at the end of the procedure. HD-based HV intervals were classified as proximal (HV_prox_), distal (HV_dis_), and right bundle (HV_RB_) recordings. Conduction velocities and sex-specific differences were analyzed.

**Results:**

A total of 67 patients (mean age 64 ± 10 years, 33%women) were evaluated. The clinical HV interval was 45 ± 9 ms and shorter in women (40 ms vs. 47 ms, *p* < 0.001). HV_prox_ was longer than HV_dis_ (51 ± 10 ms vs. 46 ± 10 ms, *p* < 0.001) and HV_RB_ (37 ± 10 ms; *p* < 0.001). Abnormal HV intervals (> 55 ms) were more often detected using HV_prox_ than HV_dis_ or HV_RB_. Conduction velocities did not differ by sex and correlated poorly with surface ECG parameters.

**Conclusion:**

HV interval measurements vary significantly by recording site and sex. Proximal measurements reveal longer HV intervals and detect more conduction delays, while women have systematically shorter HV intervals. Anatomical precision and sex-specific interpretation are important for accurate diagnosis of infranodal conduction delay.

**Graphical Abstract:**

Abbreviations: Dis: Distal His-Ventricle measurement, Prox: Proximal His-Ventricle measurement, RB: His-ventricle measurement over the right bundle.

**Figure:**
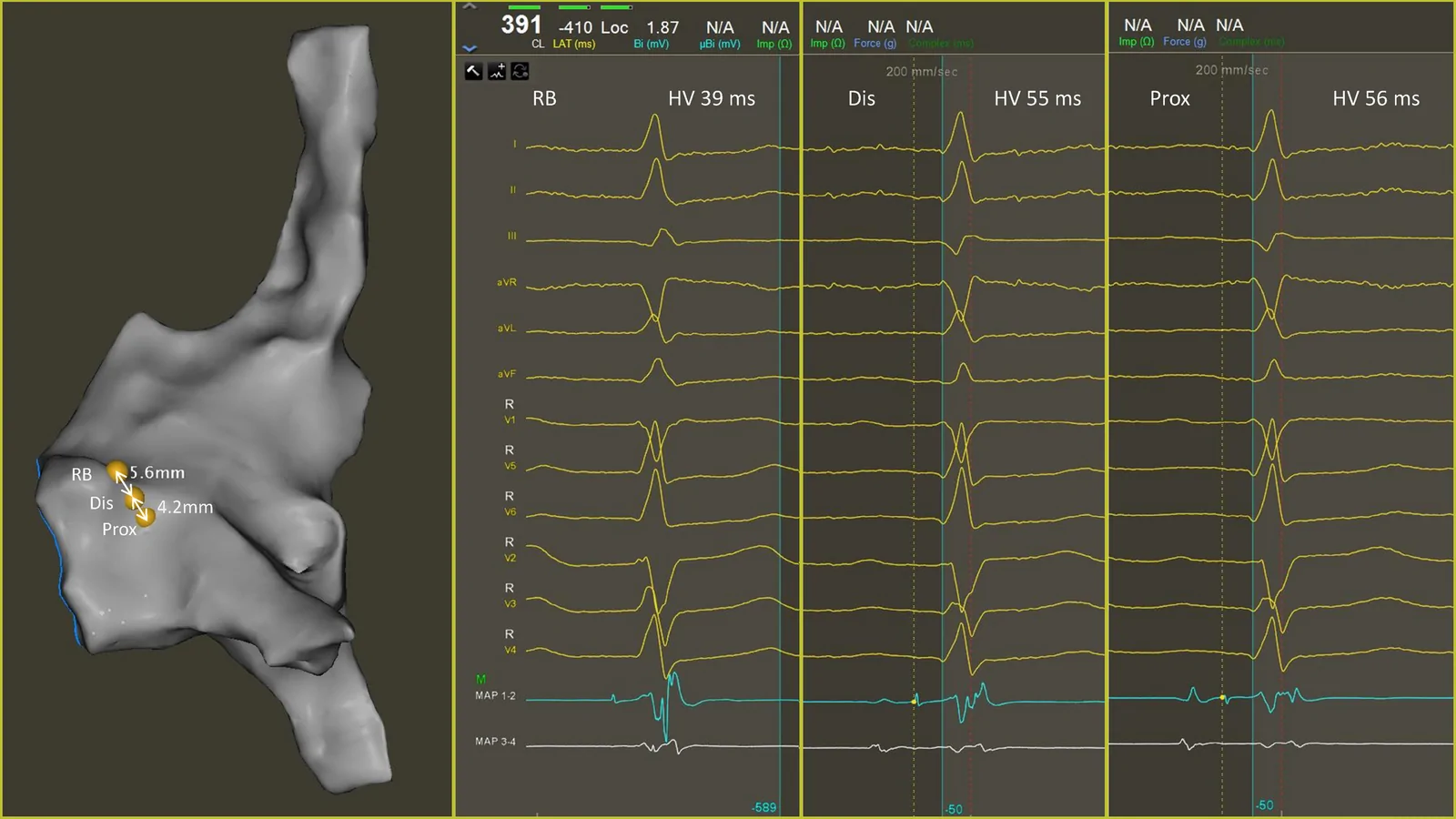

**Supplementary Information:**

The online version contains supplementary material available at https://doi.org/10.1007/s10840-026-02438-z.

## What is new?

HV interval measurements differ significantly by recording site, with proximal high-density mapping catheter recordings yielding longer intervals and identifying more patients with abnormal conduction than distal recordings.

Women exhibit consistently shorter HV intervals than men, across all measurement locations, emphasizing the need for sex-specific reference values in conduction assessment.

Standard clinical HV measurements closely reflect distal values, potentially underestimating conduction delay and influencing pacemaker decision-making.

**Supplementary Information:**

The online version contains supplementary material available at https://doi.org/10.1007/s10840-026-02438-z.

## Introduction

Accurate evaluation of infrahisian conduction is crucial for risk stratification in patients with suspected infranodal conduction disease across various clinical scenarios. The 2021 ESC Guidelines on Cardiac Pacing [1] recommend measurement of the His–ventricular (HV) interval in patients with unexplained syncope and bundle branch block, neuromuscular disorders with conduction abnormalities, or persistent conduction disturbances following TAVI, to detect infranodal conduction delay and inform decisions regarding permanent pacemaker implantation.

According to current guidelines, an HV interval < 55 ms is considered normal, > 55 ms abnormal, and ≥ 70 ms as typically warranting pacemaker implantation, especially in symptomatic individuals or those with high-risk features [2, 3]. Nonetheless, several studies reported that these cut-offs may not account for sex differences as women were shown to have shorter HV intervals than men [4–8].

Following the protocols of Josephson [9] and Zipes [10], the HV interval is measured from the earliest His bundle deflection to the onset of the QRS complex on the surface ECG. Ideally, the initial His electrogram deflection is identified by a prominent atrial electrogram; however, a prominent His deflection accompanied by a smaller atrial signal may lead to underestimation of the HV interval [11].

Although HV interval measurements can be obtained with standard quadripolar or ablation catheters, capturing the most proximal His signal is often technically demanding and time-consuming. High-density (HD) mapping catheters now allow detailed electroanatomical mapping through acquisition of large numbers of mapping points. In clinical practice, signals consistent with His electrograms are often annotated across different anatomical sites, forming a so-called "His-cloud," for instance to guide safe ablation distances. However, scientific data systematically evaluating the variation in HV interval across these His-cloud recordings are scarce.

In this study, we sought to investigate the impact of recording site and sex on HV interval measurements using a HD mapping catheter.

## Methods

### His-interval measurement

We included consecutive patients undergoing catheter ablation for atrial fibrillation (AF) between September 2021 and June 2023. At the end of the ablation procedure, all patients received additional His-Ventricle interval (HV) measurements using both a decapolar and a high-density mapping catheter. The HV interval was measured using a decapolar catheters with 2 mm interelectrode spacing (Biosense Webster, USA) and a multipolar high-density mapping catheter (Pentaray or Octaray) at the end of the procedure in sinus rhythm using the 3D electroanatomic local activation map (Carto 3, Biosense Webster, USA). For signal filtering, a Low Pass of 300 Hz and a High Pass of 30 Hz with a notch filter at 50 Hz were used. A sample size of at least 50 patients was targeted based on prior literature for agreement assessment (intraclass correlation coefficient).

All HV measurements were defined as the time between the onset of the intracardiac His deflection and the onset of the QRS interval on the 12-lead surface ECG with a sweep speed of 100 mm/s. The HV interval was measured with the high density mapping catheters using three different methods based on the His Bundle electrogram – a proximal measurement (HV_prox_ defined by a large A signal, a His signal and a small V signal, see main Figure), a distal measurement (HV_dis_ defined by a small A signal, a His signal and a V signal) and a right-bundle measurement (HV_RB_ defined as an electrogram without A signal, a His Signal and a large V signal). These measurements were then compared to the clinical HV interval measurement using a decapolar catheter. All measurements were performed at the end of the procedure in sinus rhythm.

The conduction speed through the various segments of the His bundle was calculated using the distance between the measurement locations and the difference in conduction time of the 3D electroanatomic activation map.

Finally, the agreement in HV interval measurements was calculated, between the HV_prox_ from the high-density mapping catheter and the intraprocedural clinical HV measurement.

All measurements and tests were evaluated for male and female participants separately.

### Statistical methodology

Continuous variables were summarized by means (standard deviation – SD) or medians (interquartile range – [IQR]) and were subjected to appropriate statistical tests based on the data distribution. Specifically, the Student's t-test and Mann–Whitney U test were utilized for comparisons, depending on the data's distribution characteristics. Paired data was assessed using either the paired versions of the t-test or the Wilcoxon signed-rank test, contingent upon the data distribution.

Categorical data underwent evaluation employing the Chi-squared test or Fischer test, as deemed suitable. Correlation analyses employed either Pearson's or Spearman's correlation coefficient, depending on the data distribution. Two complementary models were used. First, a multivariable linear regression analyzed the clinical (decapolar) HV interval — a single measurement per patient — adjusted for prespecified covariables (age, sex, coronary heart disease, hypertension, diabetes, body surface area, atrial fibrillation type, and rhythm at presentation); the outcome was log-transformed due to right-skew, and coefficients are reported as exponentiated (relative) effects. Second, because each patient contributed four HV measurements (clinical, proximal, distal and right bundle), the comparison across recording sites was performed with a linear mixed-effects model including a random intercept per patient to account for the within-patient correlation of repeated measurements; recording site was entered as a four-level fixed factor (clinical HV as reference), adjusted for the same prespecified covariables.

Sex-specific effects were assessed by including sex × recording-site (for the HV interval) and sex × segment (for conduction velocity) interaction terms in the mixed-effects models.

Measurement consistency with a decapolar catheter and the multipolar catheter in measuring the HV interval was assessed using the intraclass correlation coefficient with a two-way random-effects model, employing the average of all measurements and by Pearson’s R and R squared. The results were visually depicted through a Bland–Altman plot. Statistical significance was established at a P-value of 0.05. All statistical analyses were conducted using R version 4.2.1 (Vienna, Austria).

## Results

A total of 67 patients (mean age 64 ± 10 years, 33% women) were evaluated. An overview of the baseline characteristics is presented in Table 1. Compared with men, women had lower rates of hypertension, shorter P-wave duration on surface ECG, and a different distribution of atrial fibrillation.

**Table 1:** Baseline Characteristics

Variable	Overall, N = 67^*1*^	Sex		p-value^*2*^
		female, N = 22^*1*^	male, N = 45^*1*^	
Age, years	64 (10)	66 (12)	63 (10)	0.33
Body Surface Area, m^2^	2.1 (0.2)	1.9 (0.2)	2.2 (0.2)	< 0.001
Arterial Hypertension, n%	42 (63%)	9 (41%)	33 (73%)	0.010
Diabetes, n%	7 (10%)	1 (4.5%)	6 (13%)	0.41
Coronary Heart Disease, n%	13 (19%)	2 (9.1%)	11 (24%)	0.19
Chronic Kidney Disease, n%	6 (9.0%)	1 (4.5%)	5 (11%)	0.66
Hyperthyroidism, n%	2 (3.0%)	1 (4.5%)	1 (2.2%)	> 0.99
Hypothyroidism, n%	6 (9.0%)	3 (14%)	3 (6.7%)	0.39
Atrial Flutter, n%	7 (10%)	1 (4.5%)	6 (13%)	0.41
Paroxysmal Atrial Fibrillation, n%	27 (41%)	15 (68%)	12 (27%)	0.001
CHA2DS2–VA Score	2 (1.6)	1.8 (1.6)	2.1 (1.6)	0.077
Rhythm Before EP Study				< 0.001
Atrial Fibrillation, n%	25 (37%)	2 (9.1%)	23 (51%)	
Atrial Flutter, n%	2 (3.0%)	0 (0%)	2 (4.4%)	
Sinus Rhythm, n%	40 (60%)	20 (91%)	20 (44%)	
P Wave Duration, ms	125 (27)	115 (27)	131 (26)	0.033
PR Interval, ms	168 [133–205]	148 [128–174]	179 [143–206]	0.055
QRS Duration, ms	100 [94–150]	95 [90–142]	102 [94–152]	0.15
QT Interval, ms	410 [382–438]	420 [386–440]	410 [380–434]	0.82
Class I c AAD	3 (4.5%)	1 (4.5%)	2 (4.5%)	0.99
Class II AAD	43 (65%)	14 (64%)	29 (66%)	0.99
Class III AAD	13 (20%)	3 (14%)	10 (23%)	0.52

^*1*^Mean (SD), Median [IQR] or Frequency (%) ^*2*^Welch Two Sample t-test; Pearson's Chi-squared test; Fisher's exact test; Wilcoxon rank sum test

Abbreviations: AAD – antiarrhythmic drug, EP – electrophysiological, IQR – Interquartile range, SD – standard deviation

The HV interval measured with a decapolar catheter (clinical HV interval) was 45 ± 9 ms overall, but significantly shorter in women than in men (40 ms vs 47 ms, p < 0.001). This sex difference was attenuated after multivariable adjustment for age, body surface area, coronary artery disease, diabetes, hypertension, type of atrial fibrillation and rhythm at presentation (p = 0.04).

### His-cloud measurements

Using the HD mapping catheter, the mean proximal HV (HV_prox_) was 51 ± 10 ms, with significantly shorter values in women compared to men (48 ± 9 ms vs. 53 ± 10 ms, p = 0.03). The mean distal HV interval (HV_dis_) was 46 ± 10 ms (42 ± 8 ms in women vs. 48 ± 10 ms in men, p = 0.005). On average, HV_prox_ exceeded HV_dis_ by 5.1 ± 3 ms (women: 5.9 ± 3.5 ms; men: 4.7 ± 2.6 ms, p = 0.15) (Table 2).

**Table 2:** His-Cloud Measurements

Variable	Overall, N = 67^*1*^	Sex		p-value^*2*^
		female, N = 22^*1*^	male, N = 45^*1*^	
Proximal HV Interval, ms	51 (10)	48 (9)	53 (10)	0.034
Distal HV Interval, ms	46 (10)	42 (8)	48 (10)	0.005
HV with Right Bundle Signal, ms	37 (10)	33 (8)	40 (10)	0.002
HV Delta Proximal–Distal, ms	5.1 (3.0)	5.9 (3.5)	4.7 (2.6)	0.15
HV Delta Distal-RB, ms	8.4 (5.1)	8.9 (5.5)	8.2 (4.9)	0.65
Overall Propagation Speed, m/s	1.5 (0.6)	1.4 (0.6)	1.5 (0.6)	0.59
Proximal Propagation Speed, m/s	1.8 (1.0)	1.6 (0.9)	1.8 (1.0)	0.43
Distal Propagation Speed, m/s	1.4 (0.7)	1.5 (0.8)	1.4 (0.6)	0.61

^*1*^Mean (SD), Median [IQR] or Frequency (%) ^*2*^Welch Two Sample t-test

Abbreviations: HV- His-ventricle, RB – right bundle, SD – standard deviation

**Fig. 1:**
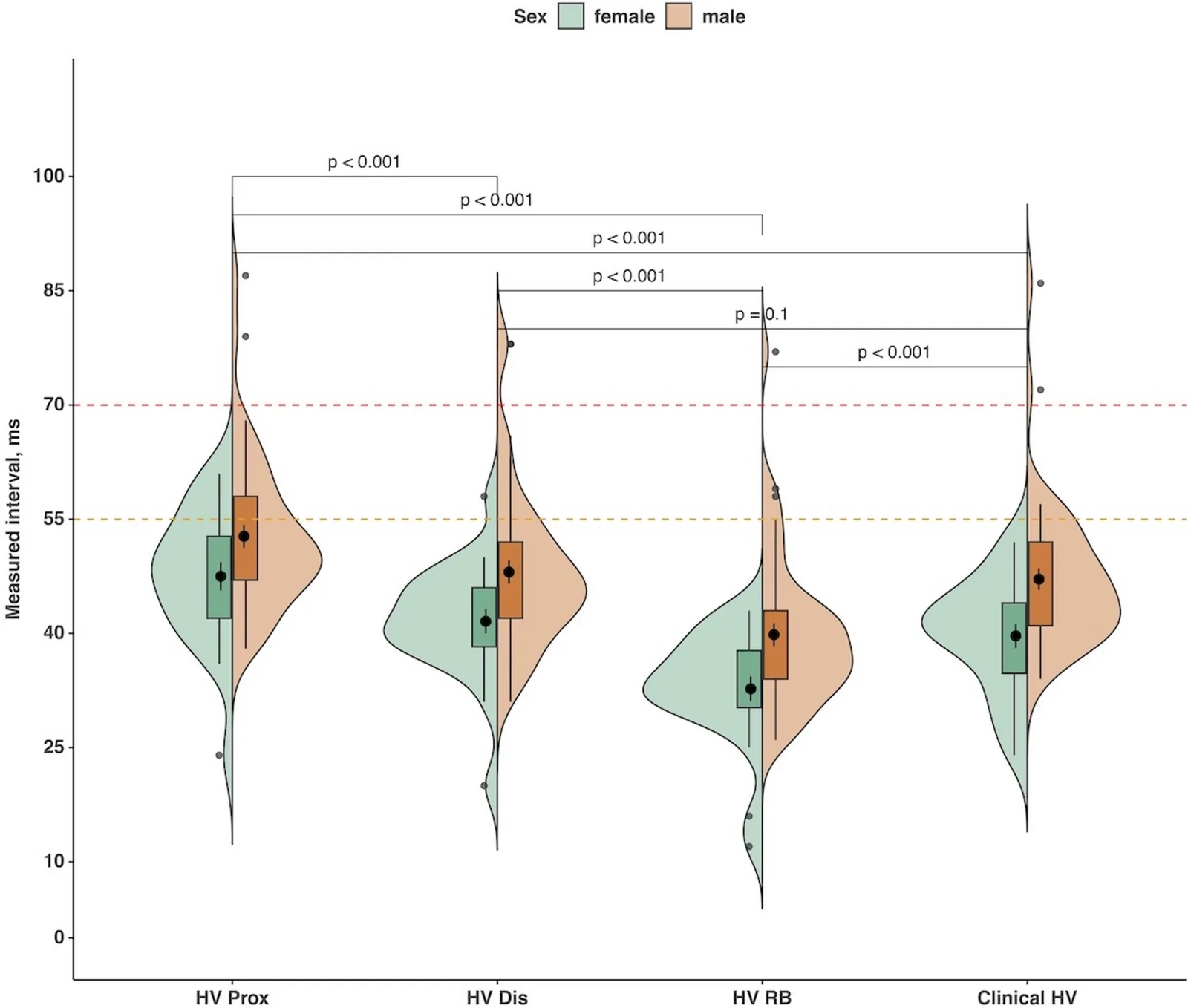
Split Violin Plot of the HV interval in female (green) vs male(orange) patients in various catheter mapping positions. Proximal HV measurement (left) are significantly longer than the distal and Right Bundle measurements. Regardless of catheter position, the HV interval is always longer in men. Abbreviations: HV – His-Ventricle, RB – Right Bundle

**Fig. 2:**
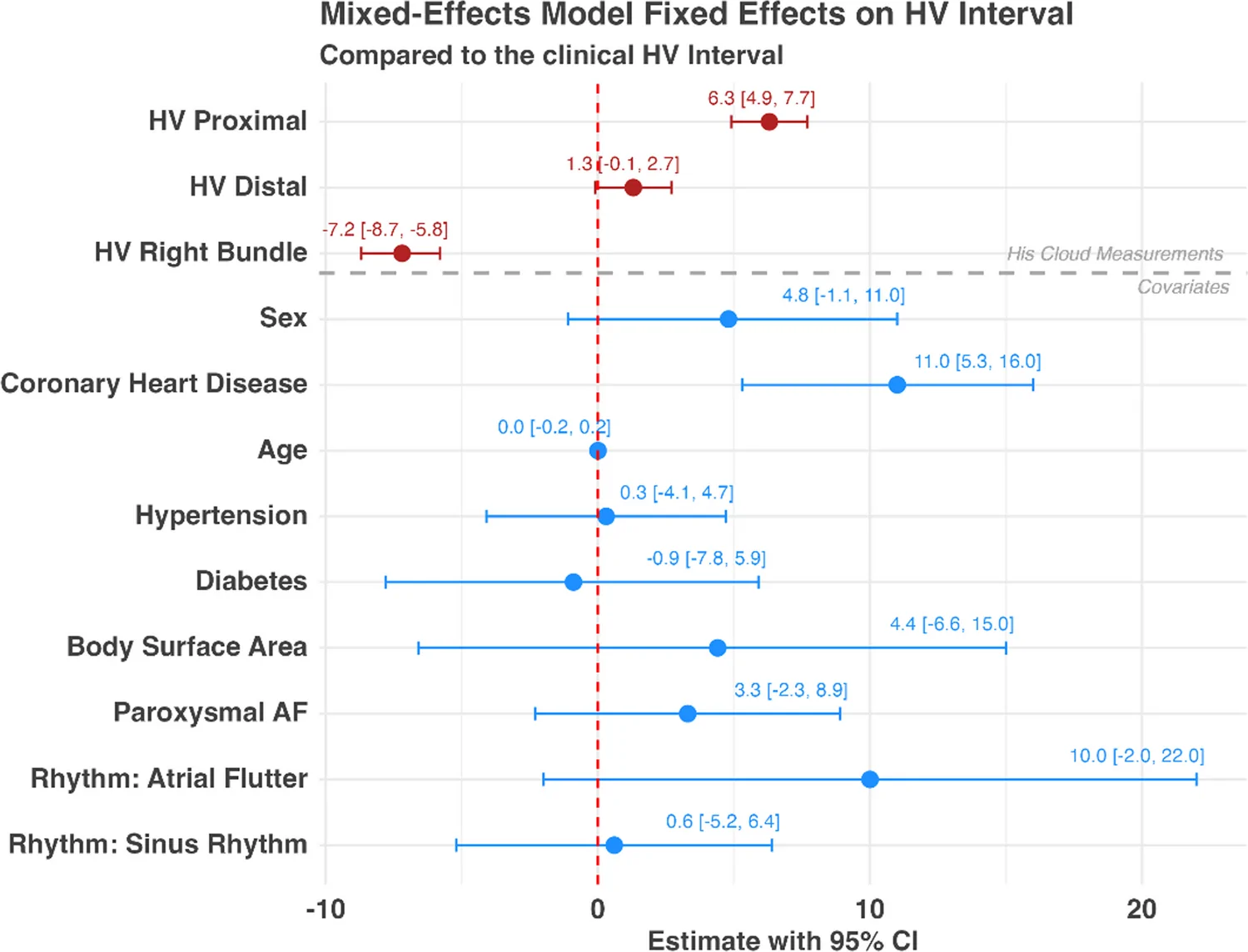
Coefficient plot from a linear mixed-effects model showing the relative effects of measurement methods and clinical covariates on HV interval, with firebrick indicating measurement method estimates compared to clinical HV interval, and black for covariate effects; error bars represent 95% confidence intervals

When measured over the right bundle (HV_RB_), the HV interval was significantly shorter, with a mean of 37 ± 10 ms (women: 33 ± 8 ms, men: 40 ± 10 ms, p = 0.002). Compared to HV_dis_, HV_RB_ was on average 8.4 ± 5.1 ms shorter (women: 8.9 ± 5.5 ms, men: 8.2 ± 4.9 ms, p = 0.65). Differences in HV interval duration by measurement site and sex are visualized in the split violin plot (Fig. 1). Using linear mixed-effects models the differences between the four types of HV interval measurements remained statistically different after multivariable adjustment for age, sex, body surface area, hypertension, coronary artery disease, diabetes, type of atrial fibrillation and rhythm at presentation (Fig. 2).

Clinically, measurement location impacted the detection of prolonged HV intervals. Using the proximal site, 19 patients (28%) had abnormal HV intervals > 55 ms, compared to 8 patients (12%) using distal measurements and only 3 patients (5%) using right bundle recordings. Notably, two patients had HV intervals ≥ 70 ms (proximal and distal). Sensitivity analyses excluding patients with thyroid disorders, as well as an analysis accounting for antiarrhythmic treatment revealed similar results.

The conventional decapolar measurement most closely matched the distal His recording (mean difference 1.3 ms) and underestimated the proximal HV by 6.3 ms (95% CI 4.8–7.9). Relative to the proximal recording, a distal signal was on average 5.1 ms shorter (95% CI 4.4–5.8) and a right-bundle signal 13.5 ms shorter (95% CI 12.2–14.9).

### His bundle propagation speed

**Fig. 3:**
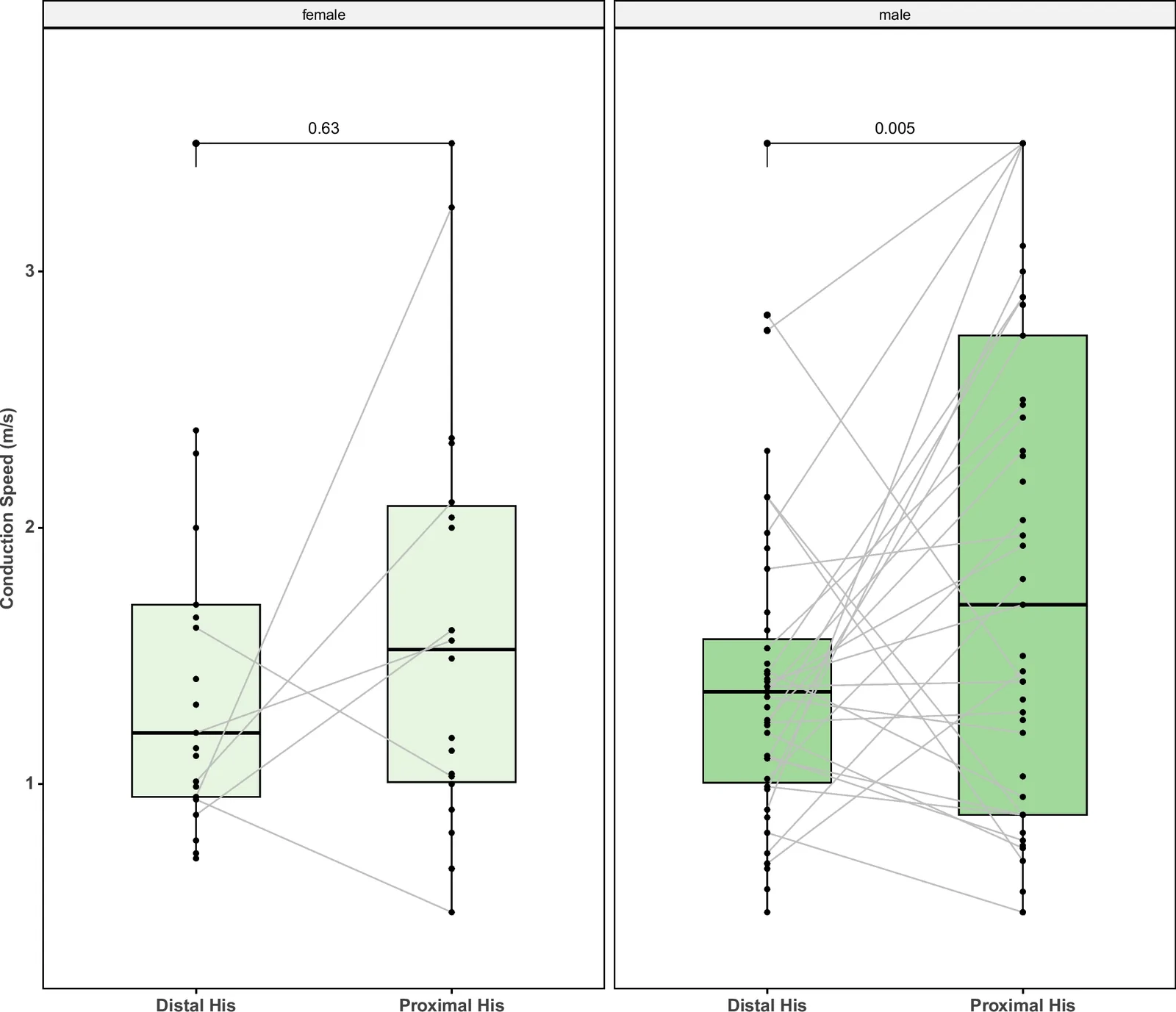
The conduction speed in the proximal portion of the His bundle seems to be higher on average compared to the distal portion. In men, this difference is significant, while in women the conduction speed is more uniform

The overall propagation speed from the proximal His recording site to the right bundle location was 1.5 ± 0.6 m/s, with no significant difference between sexes (women: 1.4 ± 0.6 m/s vs. men: 1.5 ± 0.6 m/s, p = 0.6). Sex-stratified differences in propagation times between His bundle recording sites are shown in Fig. 3.

Estimated conduction velocity between the proximal and distal His bundle locations were 1.8 ± 1.0 m/s (women: 1.6 ± 0.9 m/s, men: 1.8 ± 1.0 m/s, p = 0.43). From the distal His to the right bundle, propagation speed was slightly lower at 1.4 ± 0.7 m/s (women: 1.5 ± 0.8 m/s, men: 1.4 ± 0.6 m/s, p = 0.6). In men, the distal conduction velocity was significantly slower than the proximal (1.4 ± 0.6 m/s vs. 1.8 ± 1.0 m/s, p = 0.005), whereas in women no significant difference was observed (1.5 ± 0.8 m/s vs. 1.6 ± 0.9 m/s, p = 0.63), however the interactions between sex and conduction velocity overall and within segments were not statistically significant (Supplement).

Correlation analysis revealed that overall, His propagation speed showed weak correlation with the surface ECG: P wave duration (R = –0.30, p = 0.02), PR interval (R = –0.23, p = 0.07), QRS duration (R = 0.20, p = 0.14), QT interval (R = 0.05, p = 0.70). No other baseline clinical parameters were associated with propagation speed.

### Agreement in HV interval measurements

**Fig. 4:**
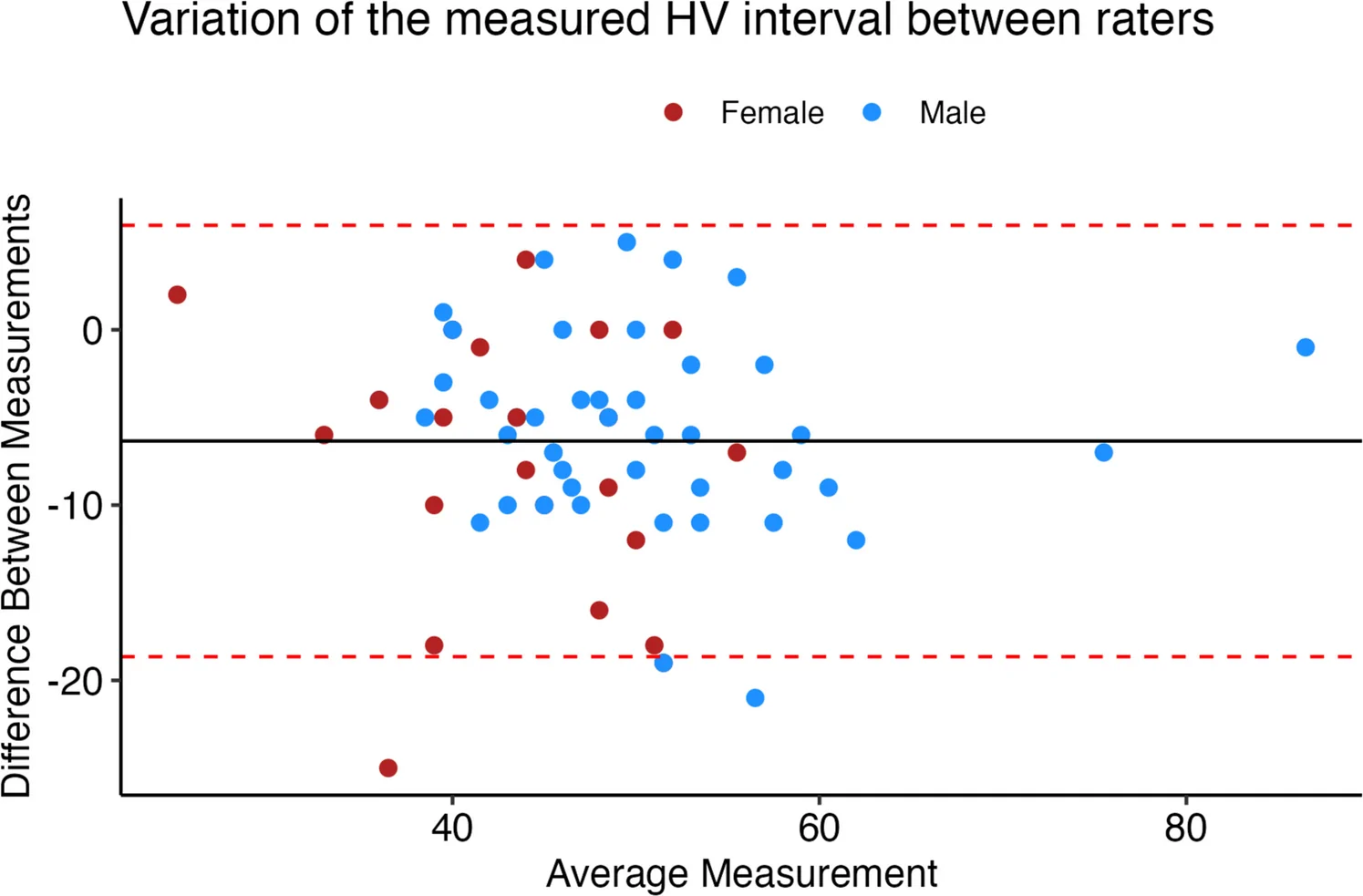
Bland Altmann Plot showing the variation of the HV interval measurements between the two catheters dependent on sex

The proximal HV was measured in all patients with two catheters, a decapolar catheter and a high-density mapping catheter. The mean HV interval measured 45 ± 9 ms using a decapolar catheter (i.e., the clinical HV measurement) and 51 ± 10 ms with a high-density mapping catheter, respectively (mean difference -6 ms, 95% limits of agreement -19, 6 ms, p_paired T-test_ < 0.001). In women, the mean difference in measurements were slightly higher at -8 ms vs -6 ms in male (p = 0.18). Male patients demonstrated excellent agreement between methods (ICC 0.92; 95% CI, 0.85—0.95), while in female patients the agreement was good (ICC 0.72; 95% CI, 0.33—0.88). In patients with cardiovascular risk factors, agreement between the two measurement methods was excellent (ICC = 0.91; 95% CI, 0.85—0.95) but was moderate in case of patients without cardiovascular risk factors (ICC = 0.54; 95% CI, –0.13—0.82). The differences between measurements are illustrated in the Bland-Altmann plot (Fig. 4). Pearson’s correlation coefficient was R = 0.79 and the reliability coefficient was R^2^ = 0.62, p < 0.001.

## Discussion

In this study, we systematically evaluated the variability of HV interval measurements using a HD mapping catheter, focusing on the impact of measurement location and sex. We found that proximal HV intervals, in accordance with the definitions by Josephson [9] and Zipes [10], were on average 11% longer than distal HV intervals and 38% longer than right bundle measurements. Notably, the clinical HV interval recorded in clinical practice, closely mirrored the distal measurement, suggesting that standard HV interval measurements may underestimate conduction delay, especially when measured distally. Abnormal HV intervals > 55 ms were measured in 28% using proximal recording compared with only 12% using distal recordings, underscoring the importance of proximal HV measurements to avoid misclassification of conduction disease.

Several additional observations emerged from our analysis: First, we observed important sex-specific differences: Across all measurement sites (proximal, distal, and over the right bundle) women consistently exhibited shorter HV intervals than men. While overall His-bundle propagation velocity was similar between sexes, men demonstrated a significant drop in conduction speed from proximal to distal, a finding requiring further validation in larger cohorts. Second, His-bundle propagation velocity correlated poorly with any of the ECG parameters and baseline parameters with only the P wave duration showing a modest correlation (R = –0.30, p = 0.02).

Our findings emphasize the importance of both sex-specific reference values and the anatomical measurement site in accurately diagnosing His–Purkinje conduction disease. If the HV interval is measured too distally, clinically relevant conduction delays may be overlooked, especially in women who inherently present with shorter HV intervals [4, 5]. This observation aligns with earlier studies suggesting potential sex-related differences in cardiac conduction [4, 5, 12–14], possibly driven by variations in heart size [15], myocardial fibrosis, or ion channel distribution [6, 7]. Because women exhibit a systematically lower baseline HV, a single fixed 55 ms cutoff represents a higher relative threshold in women and may under-detect infranodal delay in women. Consistent with this, no woman in our cohort reached the pacemaker-associated threshold of 70 ms at any recording site, and women exceeded 55 ms only with proximal recording (23%). Whether sex-adjusted reference values would alter pacemaker decision-making remains unclear.

The HV interval may also come with a risk of overdiagnosis: proximal HV interval measurements, while sensitive, may inflate the prevalence of abnormal findings, potentially prompting unwarranted pacemaker implantation in borderline cases [16–18]. The clinical implications of overdiagnosis are substantial, as pacemaker therapy, although effective, carries non-negligible risks, including lead dislodgement, perforation, infection and is associated with a higher overall mortality [19–22]. Thus, a balanced interpretation integrating clinical and electrophysiological data remains essential.

As a single fixed threshold has not been validated across recording sites and the definition of the HV interval implies a proximal measurement, applying the conventional cutoff to more distal recordings underestimates conduction delay. As the proximal signal may be difficult to obtain in routine practice, an electrogram-based correction toward the proximal value — approximately + 5 ms for a distal signal and + 13 ms for a right-bundle signal — may serve as a pragmatic alternative when high-density His-cloud mapping is not available or is too resource-intensive. Nonetheless, such an approach requires further validation in larger cohorts of patients.

The agreement between the two measurements showed good overall reliability in HV interval measurements, but with greater variability among patients without cardiovascular risk factors. This suggests that measurement consistency is impacted by patient characteristics, reinforcing the need for standardized approaches.

## Limitations

This study has several limitations. It included only patients undergoing catheter ablation for atrial fibrillation, which may limit generalizability to the general population as atrial fibrillation may be associated with underlying atrial disease, autonomic dysfunction, structural remodelling. Propagation speed calculations relied on estimated distances and did not fully account for the 3D anatomy of Koch’s triangle. No pharmacological or pacing stress protocols were applied to assess dynamic changes in conduction. The low number of females in the study may limit the power of some sex-specific analyses and may lead to model overfitting.

## Conclusion

HV interval measurements vary significantly by catheter position and sex, with proximal recordings yielding consistently longer intervals and with women showing shorter HV intervals than men across all sites. Clinicians should incorporate anatomical location, sex-specific differences, and clinical context when interpreting HV intervals. Future research should focus on standardizing HV measurement protocols to optimize diagnostic accuracy and minimize both under- and overtreatment.

## Supplementary Information

Below is the link to the electronic supplementary material.

Supplementary file1 (DOCX 23 KB)

## Data Availability

No datasets were generated or analysed during the current study.
