# Supplementary material for: High-density mapping of his-ventricular intervals: impact of recording site and sex-specific differences
Source: J Interv Card Electrophysiol. 2026 Sep 18;69(6):1493–501. doi: 10.1007/s10840-026-02438-z (PMC13645979; doi:10.1007/s10840-026-02438-z)
Supplement: Supplementary file 1 [file 10840_2026_2438_MOESM1_ESM.docx]

**High-density Mapping of His-Ventricular Intervals:**

**Impact of Recording Site and Sex-specific Differences**

-Supplement-

**Methods**

Mixed-effects model specification. HV interval values from the four recording methods were stacked into long format (four observations per patient). We fitted the linear mixed-effects model

HVᵢⱼ = β₀ + bᵢ + β₁·methodᵢⱼ + γ·covariablesᵢ + εᵢⱼ

where i indexes the patient and j the recording method.

Fixed effects: method (four-level factor: clinical HV [reference], proximal, distal and RB measurements) as the term of interest, plus the prespecified patient-level covariables age, sex, coronary heart disease, hypertension, diabetes, body surface area, atrial fibrillation type, and rhythm at presentation.

Random effect: a patient-specific random intercept bᵢ ~ N(0, σ²_patient), which models the correlation among the four measurements of the same patient; εᵢⱼ ~ N(0, σ²) is the residual.

Because the covariables are constant within a patient, only the method effect is estimated within-patient (from all 268 measurements), whereas the covariable effects are estimated between-patient (across 67 patients). The method coefficients therefore represent the adjusted mean difference of each recording site relative to the clinical HV interval. The random intercept was chosen over ordinary regression on the stacked data specifically to avoid pseudoreplication, which would otherwise treat the 268 correlated measurements as independent and underestimate the standard errors.

Supplementary Table 1 – Sensitivity analysis of HV interval measurements after excluding patients with thyroid disorders

|  |  | **Sex** | |  |
| --- | --- | --- | --- | --- |
| **Variable** | **Overall**, N = 60*^1^* | **female**, N = 19*^1^* | **male**, N = 41*^1^* | **p-value***^2^* |
| Proximal HV Interval, ms | 51 (10) | 48 (9) | 53 (10) | 0.055 |
| Distal HV Interval, ms | 46 (10) | 42 (8) | 48 (11) | 0.012 |
| HV with Right Bundle Signal, ms | 38 (10) | 33 (7) | 40 (10) | 0.004 |
| HV Delta Proximal, ms | 5.1 (2.9) | 6.0 (3.2) | 4.7 (2.7) | 0.14 |
| HV Delta Distal, ms | 8.1 (4.6) | 8.4 (4.7) | 7.9 (4.7) | 0.74 |
| Overall Propagation Speed, m/s | 1.5 (0.6) | 1.4 (0.6) | 1.5 (0.6) | 0.52 |
| Proximal Propagation Speed, m/s | 1.8 (1.0) | 1.6 (0.9) | 1.9 (1.0) | 0.22 |
| Distal Propagation Speed, m/s | 1.5 (0.7) | 1.5 (0.8) | 1.4 (0.6) | 0.72 |
| *^1^* Mean (SD), Median [IQR] or Frequency (%) *^2^* Welch Two Sample t-test | | | | |

Supplementary Table 2 – Antiarrhythmic Treatment

|  |  | **Sex** | |  |
| --- | --- | --- | --- | --- |
| **Antiarrhythmic Drug Class** | **Overall**, N = 67*^1^* | **female**, N = 22*^1^* | **male**, N = 45*^1^* | **p-value***^2^* |
| Class I (any) | 3 (4.5%) | 1 (4.5%) | 2 (4.5%) | >0.99 |
| Class II (Betablocker) | 43 (65%) | 14 (64%) | 29 (66%) | >0.99 |
| Class III (Amiodarone/Sotalol) | 13 (20%) | 3 (14%) | 10 (23%) | 0.52 |
| Class Ic | 3 (4.5%) | 1 (4.5%) | 2 (4.5%) | >0.99 |
| Class Ia | 0 (0%) | 0 (0%) | 0 (0%) |  |
| Class Ib | 0 (0%) | 0 (0%) | 0 (0%) |  |
| Class IV (Ca-antagonist) | 0 (0%) | 0 (0%) | 0 (0%) |  |
| Class V | 0 (0%) | 0 (0%) | 0 (0%) |  |
| *^1^* n (%) *^2^* Fisher's exact test | | | | |

Supplementary Table 3 – Sensitivity analysis on antiarrhythmic drug treatment

|  | | | |
| --- | --- | --- | --- |
| Model | Sex effect (male), ms | 95% CI | p-value |
| Clinical HV, unadjusted | +7.5 | [+2.9, +12.0] | 0.002 |
| Clinical HV, multivariable-adjusted | +5.6 | [-0.1, +11.3] | 0.054 |
| Repeated-measures, prespecified covariables | +4.8 | [-1.1, +10.8] | 0.107 |
| Repeated-measures + drug classes | +5.2 | [-0.6, +11.1] | 0.077 |
| *Effect estimated in ms. Mixed models: random intercept per patient* | | | |

Supplementary Table 4 – Sensitivity analysis of the mixed-effects model to covariable specification

|  | | | | |
| --- | --- | --- | --- | --- |
|  | Full model | | Parsimonious model | |
|  | β (95% CI), ms | p | β (95% CI), ms | p |
| HV Proximal vs. Clinical | +6.3 (+4.9, +7.7) | <0.001 | +6.3 (+4.9, +7.7) | <0.001 |
| HV Distal vs. Clinical | +1.3 (-0.1, +2.7) | 0.067 | +1.3 (-0.1, +2.7) | 0.079 |
| HV Right Bundle vs. Clinical | -7.2 (-8.7, -5.8) | <0.001 | -7.2 (-8.6, -5.8) | <0.001 |
| Sex (male vs. female) | +4.8 (-1.1, +10.8) | 0.107 | +5.9 (+0.2, +11.7) | 0.043 |
| Full model adjusts for age, sex, coronary heart disease, hypertension, diabetes, body surface area, atrial fibrillation type, and rhythm at presentation; parsimonious model adjusts for age and body surface area only. β in milliseconds; 95% Wald confidence intervals. | | | | |
| *n* = 67 patients (267 measurements). Both models include a random intercept per patient. | | | | |

Supplementary Table 5 – Sensitivity Analysis for Interaction between sex and measurement location – Linear mixed-effects models with a random intercept per patient

|  | Estimate (95% CI) | p-value |
| --- | --- | --- |
| HV interval (ms; reference = clinical HV) | | |
| Sex (male), main effect | +5.9 (+0.2, +11.7) | 0.043 |
| Proximal × Sex | -2.2 (-5.2, +0.8) | 0.149 |
| Distal × Sex | -1.0 (-4.0, +2.0) | 0.520 |
| Right bundle × Sex | -0.3 (-3.3, +2.7) | 0.823 |
| Overall site × sex interaction (LRT) | — | 0.475 |
| Conduction velocity (m/s; reference = proximal segment) | | |
| Sex (male), main effect | +0.05 (-0.28, +0.37) | 0.777 |
| Distal segment × Sex | -0.30 (-0.89, +0.30) | 0.320 |
| Overall segment × sex interaction (LRT) | — | 0.311 |
| Main effects from additive models (site + sex + age + body surface area for the HV interval; segment + sex for velocity). 95% Wald confidence intervals; overall interaction by likelihood-ratio test. | | |
